# Biomarkers of Aging: From Function to Molecular Biology

**DOI:** 10.3390/nu8060338

**Published:** 2016-06-02

**Authors:** Karl-Heinz Wagner, David Cameron-Smith, Barbara Wessner, Bernhard Franzke

**Affiliations:** 1 Research Platform Active Ageing, University of Vienna, Althanstrasse 14, Vienna 1090, Austria; barbara.wessner@univie.ac.at (B.W.); bernhard.franzke@univie.ac.at (B.F.); 2 Department of Nutritional Sciences, Faculty of Life Sciences, University of Vienna, Althanstrasse 14, Vienna 1090, Austria; 3 Liggins Institute, University of Auckland, Private Bag 92019, Auckland 1142, New Zealand; d.cameron-smith@auckland.ac.nz; 4 Department of Sport and Exercise Physiology, Centre for Sport Science and University Sports, University of Vienna, Auf der Schmelz 6, Vienna 1150, Austria

**Keywords:** aging, biomarker, physical function, inflammaging, DNA based marker, molecular marker, miRNA

## Abstract

Aging is a major risk factor for most chronic diseases and functional impairments. Within a homogeneous age sample there is a considerable variation in the extent of disease and functional impairment risk, revealing a need for valid biomarkers to aid in characterizing the complex aging processes. The identification of biomarkers is further complicated by the diversity of biological living situations, lifestyle activities and medical treatments. Thus, there has been no identification of a single biomarker or gold standard tool that can monitor successful or healthy aging. Within this short review the current knowledge of putative biomarkers is presented, focusing on their application to the major physiological mechanisms affected by the aging process including physical capability, nutritional status, body composition, endocrine and immune function. This review emphasizes molecular and DNA-based biomarkers, as well as recent advances in other biomarkers such as microRNAs, bilirubin or advanced glycation end products.

## 1. Introduction

Aging is a natural and multi-factorial phenomenon characterized by the accumulation of degenerative processes that are in turn underpinned by multiple alterations and damage within molecular pathways. The alterations and damage ultimately compromise cell and tissue functions [1,2]. As such, aging is the most profound risk factor for almost all non-communicable diseases, including cardiovascular diseases, cancer, diabetes and neurological diseases. The proposed mechanisms that contribute to the aging process and the development of these chronic, age-associated diseases include DNA damage, alterations in gene and non-coding RNA expression, genotoxicity, oxidative stress, and the incidence of shorter telomeres [3,4,5]. Despite the many theories that have been proposed to explain the phenomenon of aging, as yet none has been able to fully explain the mechanisms that drive the fundamental process(es) of aging [6]. Indeed, the understanding of aging has increased markedly, with current knowledge highlighting the importance of “network” theories of aging [7]. Based on these integrative theories, aging is best described as a multi-factorial process involving complex interactions between biological and molecular mechanisms [6,8,9,10]. Given this, it is unlikely that any single or readily defined set of biomarkers will provide a valid measure of biological aging.

However, it remains an important goal to provide increasingly accurate measures or predictors of the onset of ill health. Conversely, and of equal importance, is the ability to characterize the maintenance of age-appropriate optimal health. The ability to distinguish between what is normal biological aging and when health is adversely compromised is an important area for which little experimental data exists. As such, there is no gold standard tool for assessing healthy aging and no single measure has yet qualified as a sensitive and specific biomarker of aging. This results in some panels of markers that are associated with survival, health at old age, frailty, age-related (multi-) morbidity or disability. More recent attempts have aimed to statistically link multiple biomarkers that operate in different physiological networks using techniques such as principal component analysis to generate a more comprehensive insight of age-related health [11,12]. These strategies, when combined with recently identified molecular and DNA based markers, have the future potential to improve the prediction of healthy aging [3,13,14]. Thus, this short review will summarize a selected panel of the most promising biomarkers of healthy aging in humans (see Table 1). The definition of a biomarker for aging follows the guidelines of the American Federation of Aging Research [15].

## 2. Physical Function and Anthropometry

Measures of physical capability remain important markers of current and future health. Objective and standardized tests of physical capability have been developed and increasingly used in population-based studies. Functional assessments for physical performance such as handgrip strength, chair stand, gait speed, timed up and go, and six-minute walk tests are frequently used for monitoring the biological aging process. A weaker grip strength, slower walking speed, less repetitions in chair stand test, and poorer standing balance performance are all associated with significantly greater mortality rates, independent of age in older community-dwelling populations. These finding have now been further confirmed with meta-analyses highlighting the strength of the association between slower walking speed and increased mortality rates [16,17]. More recent studies indicate that, in addition to grip strength and walking speed, standing balance and chair rise speed in middle-age predict mortality rates over 13 years of follow-up [18]. A weaker grip strength was also found to be associated with functional decline as assessed by self-reported difficulties performing activities of daily living (ADLs) [19].

Various studies and systematic reviews evaluating the risk for subsequent disability (by assessing ADLs) showed that older adults performing poorly in tests of physical capability are more likely to become functionally disabled [20,21]. There is also some evidence that poorer performance in grip strength, walking speed, strength of lower extremities and standing balance is associated with a higher risk for cardiovascular disease (CVD), dementia and institutionalization (as a marker for the loss of independence). In the UK Newcastle 85+ study, low handgrip strength was associated with multi-morbidity, cognitive impairment and disability [22]. Independent from the association with physical capacity, there is a significant decline in many aspects of cognitive functioning with aging, including memory processing, attention and visuo-spatial abilities, whereas some other aspects of cognitive functioning, such as vocabulary, may increase with age [23]. However, cognitive dysfunction is associated with higher risk of mortality in patients regardless of the underlying diagnosis. In this respect it has been shown that a score below 25 in the Mini-Mental State Examination is associated with the highest mortality rate [24].

Aging is also associated with body composition changes including increased body fat, reduced muscle mass, and reduced organ mass (with the exception of the heart). Higher abdominal adiposity is a risk factor for aging and for age-related diseases with the lowest mortality risk for those with waist circumferences below 94 cm for Caucasian men and 77 cm for Caucasian women. The relative risk of mortality is doubled for those with waist circumferences above 132 and 116 cm in men and women, respectively [25]. The body mass index (BMI) is a useful measure of overall adiposity since each 5 kg/m^2^ increase in BMI is associated with a 30% higher overall mortality, a 40% higher vascular mortality, and a 60%–120% higher diabetic, renal, and hepatic mortality [26]. In addition, high BMI, independent of gender and other confounding factors, is a risk factor for cognitive decline [27].

Irrespective of the definition of sarcopenia, the age-related loss of skeletal muscle mass and strength [28,29], both low muscle mass and poor muscle function are highly prevalent and important risk factors for disability and mortality in aging [30,31]. Cross-sectional and prospective studies examining the relationship between regional muscle mass and health outcomes consistently reported that a low skeletal muscle index (skeletal muscle mass/body mass expressed as percentage) is associated with an increased likelihood of functional impairment and disability [32,33].

Collectively these studies all continue to point towards the importance of physical measures, including mobility and body composition as important biomarkers of aging. Further refinement will be required, as newer imaging methodologies become commonly applied, including magnetic resonance imaging (MRI) and peripheral quantitative computer tomography for the measurement of body composition and muscle mass with improved precision. Future technologies including wearable devices will increasingly provide accurate and real-time data on movement patterns, distinguishing the onset and severity of age-related ill health.

## 3. Blood-Based Candidate Biomarkers

Most aging biomarkers measured within blood samples are related to cardiovascular function, glucose metabolism, inflammation, nutritional status, endocrinology and simply hematology. Although there are many less well understood inflammation- and hemostasis-related biomarkers of cardiovascular function, the classical, widely measured, and well-documented physiological markers of risk of cardiovascular-related diseases remain some of the strongest biomarkers of aging [34]. Systematic reviews and meta-analyses provide strong evidence that the lipid profile (including total cholesterol, low- and high-density lipoprotein cholesterol, and triglyceride concentrations) are predictors of morbidity and mortality [35,36,37,38].

Amongst the best studied aspects of immunosenescence is the age-related increase in inflammatory peptide biomarkers (interleukin (IL)-6, IL-1β, tumor necrosis factor-α (TNF-α) and C-reactive protein (CRP)), collectively termed “inflammaging” [39]. Higher plasma concentrations of inflammatory factors such as IL-6 and TNF-α have been associated with lower grip strength and gait speed in older adults, demonstrating the interconnection between immune and functional status in the elderly [40]. CRP has been related to all-cause and specific causes of mortality and IL-6 was found to be a strong predictor of mortality [41,42,43].

Measurement of inflammatory markers has been conducted in centenarians. Centenarians demonstrate fewer signs of inflammaging [11,12]. Whilst inflammatory peptides are either absent or lowered than that evident in younger cohorts, there is a corresponding increase in the levels of anti-inflammatory cytokines, such as IL-10 [44]. Importantly, much yet is to be understood with respect to the interactions between cytokines, the immune system and target organs. It is apparent that these inflammatory markers have many non-classical functions, including the modulation of metabolic functions, well beyond the classically described impact on inflammatory function.

Aging is associated with alterations in many aspects of metabolic and hormonal function, including altered expression of cellular insulin receptors and glucose transporter units in target tissues. Within these tissues there is corresponding changes in carbohydrate metabolism including decreased cellular glucose oxidation. These alterations result in a lowered glucose tolerance as measured by impaired ability to lower blood glucose after a standard glucose load. There are several measures of glucose tolerance with the clinically accepted measures for diagnosis of diabetes mellitus being the fasting and postprandial blood glucose concentration. Glycated hemoglobin, a measure of usual glucose concentrations over the preceding few months, which does not require fasting or a glucose challenge, has also been suggested as a feasible indicator of glucose metabolism [45].

Recently it was shown that markers related to red blood cells, more specifically hematocrit, hemoglobin and the red blood cell count are associated with significantly higher chances of adverse health-status measures such as multi-morbidity, cognitive impairment, disability and mortality [22]. Age-related changes in the endocrine system are very well established including a decline in the sex hormones estrogen and testosterone due to menopause and andropause and the reduced production of growth hormone and insulin-like growth factor-1 (somatopause) [46,47,48].

The more recently discovered adipokines such as adiponectin, ghrelin, leptin and visfatin are key regulators of inflammation as well as of central functions such as appetite regulation. Alterations in serum adipokine levels have been linked with an increased risk of obesity and metabolic syndrome [49]. Interestingly, the concentration of adiponectin appears to change with age and is linked with age-related health outcomes, however further research on the association between aging and adipokines is required [50].

Other hormonal changes including thyroid-stimulating hormone (TSH), free thyroxine (FT4) and triiodothyronine (FT3) have also been evaluated on their link to health outcomes in elderly, but only low FT3 levels were associated with an increased risk for morbidity and mortality [22]. These findings are consistent with other studies investigating aged populations, showing an association of low serum FT3 with reduced parameters of physical performance and muscle strength [51], as well as an increased disease-burden and mortality [51,52].

Nutrition-related parameters are diverse, although studies have tended to focus primarily on a small subset of micronutrients including the vitamins D, B12, B6 and folic acid. However, data are not convincing, with limited evidence that suppressed vitamin D levels are associated with increased overall morbidity and cognitive impairment [22,53].

Other very interesting aging markers are N-terminal pro-B-type natriuretic peptide (NT-proBNP) and cardiac troponin, both of which are highly linked to myocardial damage. Recently it has been shown that NT-proBNP, which is elevated in the presence of heart failure, is associated with multi-morbidity, cognitive impairment and mortality [22,54], which makes NT-proBNP an informative general marker of age-related myocardial dysfunction. Cardiac troponin is associated with physiological renewal or remodeling of the myocardium. It is significantly correlated to NT-proBNP [55,56]. Despite their validity as predictors for cardiac damage and cardiovascular diseases, both markers increase with age, importantly until very old age, in both male and female healthy subjects, which successfully qualifies them as biomarkers for human aging [22,57].

Despite the apparent relevance of many of these blood-borne measures longitudinal data to precisely identify the predictive value of these markers prior to the onset of ill health are scarce. There is, however, substantial evidence that the timely analysis of blood borne biomarkers should be a common feature of geriatric care, with the need to establish normative standards and appropriate age-related reference ranges.

## 4. Molecular/DNA-Based Markers—Are They Good Aging Predictors?

Common theories of aging, where age linearly correlates with and/or is caused by the accumulation of reactive oxygen species (ROS), DNA damage, mitochondrial dysfunction, impaired antioxidant defense and shortening of the telomeres [58], are well established in humans. However it has been reported that many of these markers increase up to a certain age, most commonly coinciding with the statistical life-expectancy. Thereafter, a plateau or even a decrease in the level of some of these biomarkers has been described.

Supported by the free radical theory of aging, it is widely accepted that the production of ROS by mitochondria accumulates over the lifespan leads to a state of chronic oxidative stress at old age. As antioxidant defense mechanisms and DNA repair capacity seem to be impaired in the elderly, DNA damage has been proposed to be a consequence of aging [59]. Impaired DNA stability increases the frequency of cytogenetic aberrations, which in turn is highly linked to age-related diseases such as cancer, diabetes, cardiovascular diseases and cognitive decline [60,61]. However, after linearly increasing until the age of 60–70 years [62,63], chromosomal damage tapers and the rate of damage diminished with increasing age (over 85 years) [64]. Notably, the same seems to be true for telomeres, the protective ends of the chromosomes [65]. Longer telomeres and higher telomerase activity contribute to the stability of the genome, to DNA integrity and are positively correlated with the aging process [66].

Both, the “regular” aging process and the development of chronic diseases are accompanied by increased DNA damage, chromosomal damage, and telomere shortening [59,60,63,67]. Importantly, people exceeding the statistical life-expectancy, and especially the very oldest age-groups including nonagenarians (90–99 years), centenarians (100–109 years) and super-centenarians (110 years and older), demonstrate a different picture of age-related diseases compared to study cohorts at or below life-expectancy [68]. Furthermore, an increasing amount of data suggests that chromosomal stability, DNA repair activity, and antioxidant defense capacity in successfully aged subjects is comparable to younger cohorts [3,69,70].

Taken together, very old humans seem to contradict traditional theories of aging regarding the age-related accumulation of DNA damage, genome instability and telomere shortening by demonstrating better DNA repair capacity and higher telomerase activity, even comparable to much younger cohorts. Whether the superior resilience of “successful” agers originates from hereditary factors or an outstanding healthy lifestyle remains a field for future research. Conclusively, markers of DNA integrity, genome stability, antioxidant defense or telomere length based on current evidence do not meet the criteria for a valid biomarker for aging.

## 5. Novel and Not-Established Markers

Bilirubin, the principal tetrapyrrole, bile pigment and catabolite of haem, is an emerging biomarker to monitor resistance against chronic non-communicable diseases. Mildly elevated serum bilirubin levels have been reported to be strongly associated with reduced CVD-related mortality and associated risk factors. Recent data also link bilirubin to all-cause mortality and to other chronic diseases, including cancer and type 2 diabetes mellitus. Therefore, there is evidence to suggest bilirubin as a biomarker for reduced chronic disease prevalence and for the prediction of all-cause mortality, but also as a novel biomarker for successful aging [71,72].

Advanced glycation end products (AGEs) represent further biomarkers with some potential to monitor healthy aging. Protein modifications such as the non-enzymatic protein glycation are common posttranslational modification of proteins resulting from reactions between glucose and the amino groups of proteins. This process, better known as “Maillard reaction”, leads to the formation of AGEs. Interestingly, the AGEs of long-lived proteins such as collagens and cartilage accumulate during normal aging. They are involved either directly or through interactions with the AGE-receptors in the pathophysiology of numerous age-related diseases including CVD, renal disease and neurodegeneration [73].

Metallothioneins (MTs) are low molecular weight, cysteine rich, zinc-binding proteins, which are down-regulated in older age groups [74]. MTs exert an essential role in zinc-mediated transcriptional regulation of genes involved in growth, proliferation, differentiation, and development, pathways of importance in neural function. There is experimental evidence that MTs are induced in the aging brain as a defensive mechanism to attenuate oxidative and nitrative stress. MTs may also act as free radical scavengers by inhibiting Charnoly body formation, thus contributing to protecting mitochondrial function as a mechanism of neuroprotection in the aging brain [75].

Very recently the interesting model of the “epigenetic clock” has been advanced with the analysis of peripheral blood mononuclear cells isolated from semi-supercentenarians and their offspring [76,77]. The epigenetic clock is a multivariate estimator of chronological age based on DNA methylation levels of 353 dinucleotide markers known as Cytosine phosphate Guanines (CpGs). Extent and patterns of CpGs are independently associated with chronological age and mortality [78]. Further data is required to understand whether these changes are correlative or causal in the maintenance of health with advancing age.

Epigenetic changes are but one of a number of emerging molecular markers of altered molecular function in aging that may be predictive of health status. Recent studies have examined the novel molecular marker p16^INK4a^, which is classically known for its capacity to inhibit cyclin-dependent kinase activity. Long-term p16^INK4a^ expression is a promoter of cellular senescence, a process of irreversible cell-cycle arrest and the loss of regenerative capacity. Therefore, precise regulation of p16^INK4a^ is essential to tissue homeostasis, maintaining a coordinated balance between tumor suppression and aging [79]. As yet, studies in human populations and differing cell types have yet to be conducted to provide evidence of its potential as a biomarker of healthy aging.

Finally, microRNAs (miRNAs), single-stranded and non-coding RNA molecules of 21–23 nucleotides that regulate a broad spectrum of biological activities, have been proposed as signatures of aging [80]. These small RNA molecules were initially demonstrated to contribute to aging of *C. elegans* and show differential expression levels in tissues of young and old animal models [81,82]. Most interestingly miRNAs are stable molecules even in serum and/or plasma, hence are regarded as promising markers in the clinical setting [83]. Of the many miRNAs expressed in the human genome, potential candidate analysis is focused on miR-146, miR-155, miR-21 and miR-126 [84,85,86]. These studies have been extended to demonstrate a specificity of age-related health loss, with the ability to differentiate the onset of Alzheimer’s disease and/or mild cognitive impairment from cognitively normal age-matched controls with some degree of accuracy utilizing a miRNA signature analysis [87]. Furthermore, miRNAs might also serve as circulating biomarkers for cardiovascular aging or aging-associated diseases [88], but further research needs to be conducted to evaluate their sensitivity, selectivity and potential as predictive biomarkers for discriminating successful from non-successful aging.

## 6. Conclusions

Due to the complexity of the many biological and molecular mechanisms of aging, no single biomarker will provide a valid measure of healthy aging. Based on the available evidence, there is yet to emerge any newly identified measures of molecular function that out-perform the existing lipid, peptide and hormonal biomarkers routinely analyzed in blood. Often overlooked is the value of combining such measures with the well-established markers of physical and functional parameters.

Currently many novel markers are under evaluation, made possible with new analysis technologies or greater insights into the fundamental molecular basis of the loss of functionality and onset of dysfunction at the cellular level. Thus the number of potential candidates is anticipated to grow markedly. The so-called “omics” techniques such as metabolomics, proteomics or genomics will further trigger data generation and might offer the opportunity for an unbiased systematic discovery route for novel biomarkers of aging. However, verification against health measurements will take some time [14]. It remains to be elucidated which markers will achieve the status as reliable predictors of biological aging and provide a measure of ongoing optimal health.

## Figures and Tables

**Table 1:** Summary of putative biomarkers of aging.

**Physical Function and Anthropometry**		
	**Practicability of Measurement**	**Outcome Prediction**
Walking speed	High	Mortality
Chair stand	High	Mortality
Standing balance	High	Mortality
Grip strength	High	Mortality
		CVD
		Cognitive impairment
Body Mass Index	High	Mortality
		CVD
		Cognitive decline
Waist circumference	High	Mortality
		CVD
Muscle mass	High	Mortality
**Blood-based candidate markers**		
	**Practicability of measurement**	**Outcome prediction**
Inflammation IL-6, TNF-a, CRP	moderate to high	Mortality, grip strength
Network analysis of inflammatory markers	moderate	Mortality
Glucose metabolism: HbA1c, plasma glucose	moderate	Mortality, CVD
Adipokines	moderate	Mortality (moderate prediction) Aging/frailty
Thyroid hormones	moderate	Mortality/morbidity (moderate prediction)
Vitamin D	low	Mortality/multimorbidity Cognitive impairment
NT-proBNP Troponin	moderate	Mortality/multimorbidity Cognitive impairment
**Molecular/DNA based markers**		
	**Practicability of measurement**	**Outcome prediction**
DNA/chromsomal damage	low	Aging (moderate prediction)
Telomere length	low	Mortality (moderate prediction)
DNA repair	low	-
**Novel markers**		
	**Practicability of measurement**	**Outcome prediction**
Bilirubin (mainly unconjugated bilirubin)	moderate to high	CVD, CVD-related mortality
Advanced glycation end products	low	CVD
Metallothioneins	low	Aging brain
DNA methylation	low	-
MicroRNAs	low	CVD aging (moderate prediction)

## Abbreviations

The following abbreviations are used in this manuscript:

AGEs	Advanced glycation end products
ADLs	Activities of daily living
BMI	Body mass index
CRP	C-reactive protein
CVD	Cardiovascular disease
FT3	free triiodothyronine
FT4	free thyroxin
IL	Interleukin
miRNA	microRNA
MRI	Magnetic resonance imaging
MTs	Metallothioneins
NT-pro-BNP	N-terminal pro-B-type natriuretic peptide
ROS	Reactive oxygen species
TNF-α	Tumor necrosis factor-α
TSH	Thyroid-stimulating hormone
